# WSO Action Plan for Stroke Prehospital Care: Top Two Priorities

**DOI:** 10.1111/cns.70374

**Published:** 2025-04-21

**Authors:** Renyu Liu, Jing Zhao, Amit Kandel, Siju V. Abraham, Gary A Ford, Marc Fisher, Jeyaraj Durai Pandian

**Affiliations:** ^1^ Department of Anesthesiology and Critical Care, and Neurology Perelman School of Medicine at the University of Pennsylvania Philadelphia Pennsylvania USA; ^2^ Department of Neurology Minhang Hospital Affiliated to Fudan University Shanghai China; ^3^ Department of Neurology and Emergency Medicine Jacobs School of Medicine and Biomedical Sciences, University at Buffalo Buffalo New York USA; ^4^ Department of Emergency Medicine Jubilee Mission Hospital, Medical College & Research Institute Thrissur India; ^5^ Radcliffe Department of Medicine University of Oxford Oxford UK; ^6^ Department of Neurology Beth Israel Deaconess Medical Center, Harvard University Cambridge Massachusetts USA; ^7^ Department of Neurology Christian Medical College Vellore India

**Keywords:** action awareness, diagnostic technologies, point of care, prehospital care, stroke

## Abstract

This editorial commentary describes the consensus reached by a group of experts from the World Stroke Organization regarding two top priorities to improve stroke prehospital care on the global stage. The first priority is effective stroke action awareness, and the second is the research and development of point‐of‐care prehospital diagnostic technologies.

After the 2024 Closed Door Round Table Discussion on improving stroke prehospital care globally, [1] leaders and experts from the world stroke organization (WSO) and the Coalition of Special Taskforces for Stroke (CSTS) gathered together online to discuss the top priorities and action plans in improving stroke prehospital care. The discussion was co‐organized by Dr. Renyu Liu (Co‐Chair, WSOTPC, University of Pennsylvania, USA) and Dr. Jing Zhao Co‐Chair of WSO taskforce of prehospital care (WSOTPC, Minhang Hospital, Fudan University, China), moderated by Dr. Marc Fisher, the ex‐president of WSO (Beth Israel Deaconess Medical Center, Harvard University, USA). The key participants included Dr. Jeyaraj Durai Pandian, the President of WSO (Christian Medical College, India); Dr. Steve Davis. Ex‐president of WSO (Melbourne University, Australia); Dr. Craig Anderson, the president elect of WSO (University of New South Wales, Australia; Dr. Gary Ford), the president of CSTS (University of Oxford, UK); Dr. Mayowa Ojo Owolabi (University of Ibadan, Nigeria); Dr. Amit Kandel (University at Buffalo, USA); Dr. Christian Foerch (RKH Klinikum Ludwigsburg, Germany); Dr. Xiuhai Guo, MD (Capital University, China); Hitoshi Fukuda (Kochi University Hospital, Japan); Dr. Qiuhong Ji (Nantong University, China); and some others.

## 1 Top Two Priorities

The team agreed that improving stroke prehospital care and access represents the next major frontier in acute stroke management and treatment. A consensus was reached on the two most critical priorities for advancing stroke prehospital care globally, including in low‐ and middle‐income countries (LMICs).

1. Effective Stroke Action Awareness: The first priority is to enhance public education so that people can recognize stroke symptoms promptly and take immediate action. Beyond merely increasing awareness, enabling timely “action” is more important. The WSOTPC has recently proposed replacing the term “stroke awareness” with “stroke action awareness” to emphasize the critical importance of knowing what to do when stroke symptoms or risk factors are identified [2]. The FAST acronym has worked quite well in English speaking countries; however, when adapted to other languages it may lose the meaning of “fast” after translation. Meanwhile it does not include the word “stroke,” and lacks a direct link to an emergency phone number. These shortcomings reduce its intuitive effectiveness in non‐English‐speaking contexts. Further research is required to optimize stroke awareness and action messaging globally.
2. Research and development of point‐of‐care prehospital diagnostic technologies: Triage diagnostics will likely become a cornerstone in prehospital stroke care, allowing differentiation between hemorrhagic and ischemic stroke. This distinction is crucial in determining whether a patient should receive immediate thrombolytic therapy or benefit from early intensive blood pressure lowering, a simple yet highly effective intervention that is particularly viable in LMICs [3]. While portable CT and MRI devices are under development, their high cost limits feasibility in LMICs. However, emerging point‐of‐care diagnostics, such as GFAP‐based rapid tests for intracerebral hemorrhage detection, offer a promising, cost‐effective solution [4, 5]. These tools could revolutionize stroke prehospital care by facilitating faster and more accurate diagnosis. They could potentially be a game changer in stroke prehospital care. Mobile stroke units are expanding rapidly in China and Thailand. Interestingly, the new lighter image devices with cheaper prices (e.g., portable CT with less 200 kg) and other triage diagnostics for point of care are going to make them more affordable and available.

## 2 The Action Plan

The team led by core members of WSOTPC is currently drafting a scientific statement on stroke prehospital care for LMICs, which is nearing completion. To raise stroke action awareness, the integration of social media and home video education is recommended [2]. The use of national stroke awareness initiatives such as “Stroke 120” program gave a good example in improving stroke awareness and reducing prehospital delay and ambulance usage [6, 7]. Similar programs, such as “Stroke 911” [8] in Mexico and “Stroke 112” [9] in Nigeria, are currently under evaluation.

Over the next 5–10 years, we should improve stroke neurology education for health care professionals including medical students, residents, nursing students, and emergency medical personnel. Globally, the median ratio of neurological specialists (neurologists, pediatric neurologists, and neurosurgeons) is 3.1 per 100,000 population [10]. Among the WHO regions, the European regions have 9 per 100,000 population whereas the African region and South‐East Asia region have a median of 0.1 and 0.3 per 100,000 populations, respectively [10]. Education is a critical focus area because it will improve public action awareness as well as emergency medical system (EMS) preparedness.

A national “Stroke Action Awareness” campaign is potentially the most effective way to raise awareness, especially the action awareness about stroke signs and symptoms, risk factors, the importance of immediate action, and the burden of stroke among policymakers, the public and healthcare providers. This initiative will enhance knowledge, drive policy changes, and improve early recognition, immediate actions, rescue of stroke, ultimately strengthening stroke care and prevention efforts. Recognizing that each country and even regions within a country may have unique needs, existing successful models will be analyzed to identify best practices for improving stroke action awareness in diverse linguistic and cultural settings, especially where English is not the main spoken language. As part of the action plan, developing artificial intelligence (AI) driven algorithms could help to enhance stroke recognition or detection and trigger emergency medical dispatch via immediate emergency call [11]. This may include mobile stroke unit or tele‐medicine enabled ambulances dispatch to improve effective actions and care after a potential stroke is recognized [12].

To implement research rapidly and effectively, collaboration with government agencies, universities, hospitals, and EMS providers is essential. Stakeholder meetings will be organized to facilitate translational research implementation, integration of prehospital stroke care initiatives into national healthcare strategies, and alignment with prehospital care models for other noncommunicable diseases (e.g., acute coronary syndrome).

Clinical trials are evaluating blood‐based diagnostics to exclude hemorrhagic stroke. Either private or public models of stroke prehospital care must be established with funding in a few countries.

WSO intends to continue holding meetings involving global stakeholders with dedicated sessions on prehospital care. The teams from WSOTPC and CSTS have hosted eight dedicated international meetings on stroke prehospital care. The team will continue sustaining it in the coming years in aligning with the action plan on stroke prehospital care of WSO. A critical next step is achieving a global consensus on a realistic and standardized target for prehospital stroke delay—one that is particularly applicable to LMICs.

## Conflicts of Interest

The authors declare no conflicts of interest.

## Data Availability

The authors have nothing to report.
